# Hydrogel Based Sensors for Biomedical Applications: An Updated Review

**DOI:** 10.3390/polym9080364

**Published:** 2017-08-16

**Authors:** Javad Tavakoli, Youhong Tang

**Affiliations:** 1 Medical Device Research Institute, College of Science and Engineering, Flinders University, Adelaide 5042, SA, Australia; 2 Institute for Nano Scale Science & Technology, College of Science and Engineering, Flinders University, Adelaide 5042, SA, Australia

**Keywords:** hydrogel based biosensor, hydrogel, bioreceptors, immobilization, biomedical application, transduction strategies

## Abstract

Biosensors that detect and convert biological reactions to a measurable signal have gained much attention in recent years. Between 1950 and 2017, more than 150,000 papers have been published addressing the applications of biosensors in different industries, but to the best of our knowledge and through careful screening, critical reviews that describe hydrogel based biosensors for biomedical applications are rare. This review discusses the biomedical application of hydrogel based biosensors, based on a search performed through Web of Science Core, PubMed (NLM), and Science Direct online databases for the years 2000–2017. In this review, we consider bioreceptors to be immobilized on hydrogel based biosensors, their advantages and disadvantages, and immobilization techniques. We identify the hydrogels that are most favored for this type of biosensor, as well as the predominant transduction strategies. We explain biomedical applications of hydrogel based biosensors including cell metabolite and pathogen detection, tissue engineering, wound healing, and cancer monitoring, and strategies for small biomolecules such as glucose, lactate, urea, and cholesterol detection are identified.

## 1. Introduction

**Figure 1:**
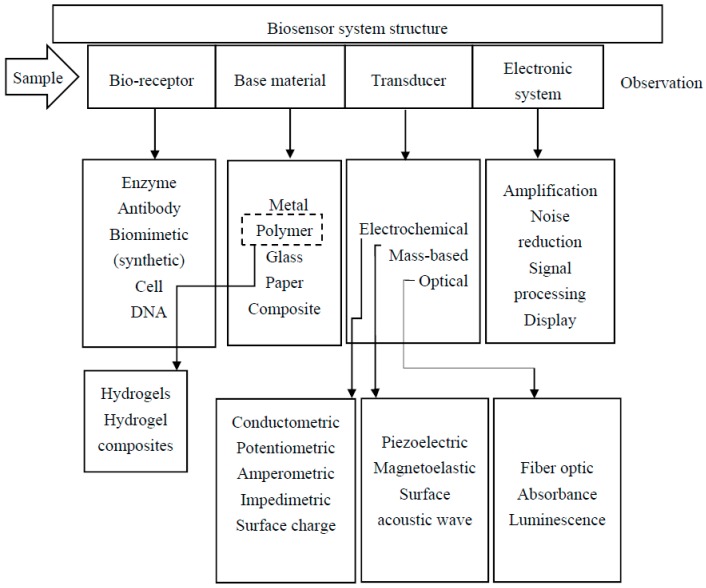
A typical biosensor system includes structural elements, materials, and strategies for sensing. Hydrogel, as a biocompatible polymer with great ability of water absorption, can be used as a base material to form a hydrogel based biosensor. Some sensing strategies (electrochemical, mass based, and optical) can be used for identification of a specific biomolecule using mentioned measurement methods (conductometric, potentiometric, amperometric, impedimetric, surface charge, piezoelectric, megnetoelastic, surface acoustic wave, fiber optic, absorbance, and luminescence). Measurement methods are not limited to the mentioned methods explained here and other classification (i.e., label based vs. label-free) can be noticed as well.

Biosensors that detect and convert biological reactions to a measurable signal have gained much attention in recent years. By integration of a biologically active component with an appropriate transducer, biosensors produce a measurable signal generated by chemical reactions. To achieve this purpose, a biosensor is made up of four main components, as shown in Figure 1.

A bioreceptor, as a molecular species—such as nucleic acid, enzyme, antibody, gene, or a biological system, such as a cell or an organ—employs a biochemical mechanism to interact with the analyte. While a transducer produces a measurable signal proportional to the bioreceptor–analyte interaction [1]. Using different immobilization techniques, bioreceptors are coupled with base materials [2]. Base materials can be made up of metals, polymers, glass, or composites. Moreover, hydrogels as a swell able polymer with enviro-sensitive properties are having a profound impact in a broad range of applications due to their exceptional physicochemical, mechanical, electrical, and optical properties [3,4,5]. Despite the period since their initial discovery in 1968, hydrogels have emerged as a promising platform with surprising and enormous potential for biomedical use. Their applications in this field are still in a developing phase [6,7,8,9,10].

In fact, searches for the word “biosensor” in the ISI Web of Science, PubMed, and Science Direct databases obtained 46,791, 47,017, and 72,477 hits respectively from the period 1950 to 1 February 2017. An advanced searches that combined “biosensors” and “hydrogel based” resulted in 702, 497, and 4047 findings in the ISI Web of Science, PubMed, and Science Direct databases. Totals of 28, 66 and 1577 papers were found for “biosensor + hydrogel based + biomedical application” searches in the ISI Web of Science, PubMed, and Science Direct databases, respectively. Through careful screening, the most published papers were revealed to be those referring to general specifications of bioreceptors and transducers. To our knowledge, however, critical reviews that describe hydrogel based biosensors for biomedical applications were rare.

The objectives of this review are to summarize the biomedical applications of hydrogel based biosensors, including bioreceptors that have been specifically recommended for hydrogels, the methods of immobilization, sensor design, and structural modifications, to identify potential roles of hydrogels for biomedical applications, and to suggest areas for future investigation.

## 2. Bioreceptors

**Figure 2:**
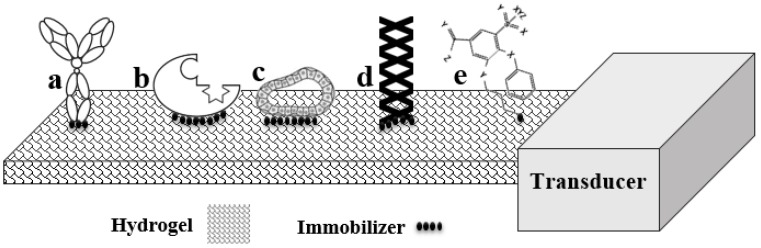
Schematic diagram of five distinct bioreceptor categories (**a**) antigen/antibodies; (**b**) enzymes; (**c**) cells and cellular structures; (**d**) nucleic acids and DNA; and (**e**) biomimetic.

Bioreceptors are biomolecular recognition components that are responsible for binding a specific particle of interest within a biosystem environment. Although many forms of bioreceptors are available to monitor numerous different particles that have been triggered for sensing, they can be categorized in five different major groups, as shown in Figure 2 [11,12].

Antibodies are Y-shaped, complex proteins used to identify foreign antigens, viruses, and bacteria. With specific binding capabilities, antibodies have been used in biosensors. The “lock and key fit”—as a unique property of a specific geometrical configuration—is the capability that an antigen-specific antibody exhibits when employed as a biosensor [13]. Exploitation of an animal immune system is a usual method for production of polyclonal, monoclonal, and recombinant antibodies, among which, polyclonal antibodies are popular for their frequent use as immune sensors [14]. Recombinant antibodies have been selected to detect structurally diverse antigens including haptens, proteins, and carbohydrate moieties.

Enzymes are another type of bioreceptor attracting attention for their specific binding capabilities and catalytic activities that amplify detection [15]. The activities are bestowed by the enzyme amino acid residues. For some of these capabilities, however, some enzymes require a cofactor, such as inorganic ions or coenzymes, e.g., complex metalorganic/organic molecules [16]. The regulatory nature of enzymes has made them attractive for use as biosensors to quantify catalytic reactions, including heat, light, and charges, i.e., protons and electrons [17]. Regulation changes of the enzymes’ selective conformation serve as a biorecognition method in biosensors [18].

Nucleic acids use the pairs of C:G (cytosine:guanosin) and A:T (adenine:thymine) in DNA for specificity of biorecognition. Hybridization of a certain sequence of a DNA molecule to a specific labeled molecule can be utilized in biosensors [19]. In fact, the DNA portion with the specific sequence is immobilized to the base material of the biosensor and facilitates biosensing upon interaction with the complementary sequence that exists on the targeted molecule [20]. Nucleic acid ligands known as aptamers are included in a nucleic acid bioreceptor’s family that is isolated from libraries of oligonucleotides through an in vitro “systematic evolution of ligands” process [21,22]. In contrast to nucleic acids, these short, single-strand oligonucleotides are believed to distinguish their targets by shape rather than by sequence. Therefore, a wide range of small molecules, cells, and proteins can be detected by using aptamer bioreceptors [23]. Within these families, catalytic aptamers —“aptazymes”—have been introduced, that have high capability of recognizing metabolic intermediates at a very low concentration [24]. Aptazymes, like enzyme bioreceptors, can be denatured frequently without loss of their catalytic or binding capabilities, and their relatively high signal-to-noise ratios make them more attractive than enzymes [25].

For biorecognition by cells or cellular structures (microorganisms), bioreceptors can be performed by either a specific cellular structure or an entire cell binding to certain species. As certain chemicals are taken up by cellular structures for digestion, a kind of chemical-specific biosensor is fashioned [26,27]. Bacteria and fungi microorganisms are categorized in the cell bioreceptor’s group and have been used for toxicity detection [28]. In the bioreceptors of a cellular structure, abundant carrier proteins that provide facilities to transport a chemical compound from one place to another (surfaces to internal parts or between cells) can be used for molecular recognition [29,30,31].

**Figure 3:**
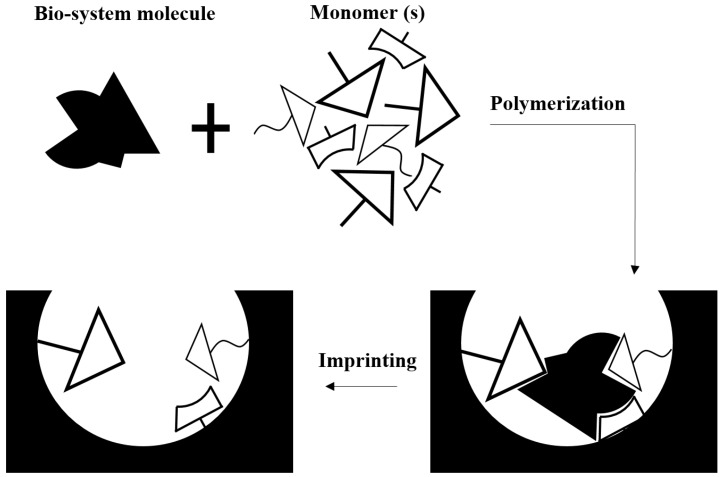
The molecular imprinting method for biosensor fabrication is performed by polymerization of biosystem molecules and monomer(s) mixtures including a high concentration of a crosslinking agent. Following polymerization and extraction of molecules, molecular holes are employed as complementary sites for the biosystem’s selected molecule.

Biomimetic receptors are artificial fabrications employed to mimic a bioreceptor [32,33]. Methods used for this purpose include artificial membrane production [34,35,36], using genetically engineered molecules such as lectin based or peptide nucleic acid based molecules [37,38,39], and molecular imprinting [40,41]. Among them, the molecular imprinting technique, which consists of mixing biomolecules with monomers and a crosslinking agent, has attracted much attention [42,43,44]. With this method, selective binding sites based on molecular templates are introduced to synthetic polymers. Figure 3 gives a schematic representation of the fabrication of a molecular imprint biosensor. Advantages and limitations of different bioreceptors are listed in Table 1.

## 3. Hydrogels for Biosensing

As water swellable three-dimensional structures, hydrogels are formed by chemical (covalent bonds) or physical (non-covalent interactions) crosslinking. These smart materials with excellent biocompatibility are considered to interface progressively with biosystems, but suffer from side effects [51,52,53]. Many features have made them popular for biosensing applications: interaction with biological components at the molecular level; their regulating viscoelastic properties; being reactive to external stimuli; possessing antifouling characteristics; and the existence of a wide range of well-known synthesis methods for incorporating bioreceptors into their highly wet structure.

Hydrogel-based biosensors can detect biological events in two ways. The first includes hydrogels without bioreceptors, whose swelling properties change in response to selected biological interactions [54]. Ionic hydrogels with environmental sensitivity—for example to pH, temperature, and electrical field—have been widely used in this group [55]. pH-sensitive hydrogels either admit or release protons in response to appropriate ionic strength alterations in the surrounding aqueous biosystem. The more ionized ionic hydrogels are, the more electrostatic repulsion between polymeric chains will be created, leading to a negative or positive swelling ratio [56]. The same scenario has been observed for polyelectrolyte gels in response to an electric field, the intensity of which was responsible for swelling and de-swelling processes. The higher the electric field intensity, the more fixed charges will exist that affect the degree of swelling [57]. A copolymer of hydrophobic and hydrophilic monomers can introduce a thermo-responsive hydrogel, where the ratio of the monomers is responsible for phase transition in response to changes in biosystem temperature [58]. Apart from the reasons mentioned for phase transition, the biological interactions in a biosystem cause swelling alteration and may translate to a macroscopic response. Hence, the macroscopic response needs to be used as optical, conductometric, amperometric, or mechanical readouts for biosensing.

With respect to hydrogel porous structures and their unique large internal surfaces, the second way of detecting biological events involves hydrogel-based biosensors that can accommodate bioreceptors for detection of biochemical or biological interactions. With this method, hydrogel immobilized bioreceptors are employed for hosting biomolecular recognition components to identify a definite event of a biosystem. Of major importance for designing this type of biosensor are stable immobilization of bioreceptors, surface bonding strategies, prevention of nonspecific protein adsorption to the hydrogel surface, probe density, flexibility, and swelling kinetics.

### 3.1. Polyvinyl Alcohol

Polyvinyl alcohol (PVA) hydrogels have been widely used in various biomedical applications due to their biocompatibility, hydrophilic properties, and biomechanical characteristics. Relying on their physical crosslinking, the performance PVA and its composites with glucose [59,60,61,62,63,64,65], triglyceride [66,67], ethanol [68], urea [69,70,71,72], hydrogen peroxide [73,74], toxicity [75,76,77], and genetic [78] sensors have been investigated. The flexibility of PVA hydrogels as well as their stability under a wide range of environmental conditions allows them to mimic soft tissue and to minimize inflammation and fibrosis, an ability that is necessary for implantable sensors.

### 3.2. Polyethylene Glycol

Polyethylene glycol (PEG), a hydrophilic biomaterial, has excellent antifouling properties conferred by its low interfacial energy, resisting protein and cell surface adhesion [79,80,81]. With its biocompatibility, PEG is widely used as a biosensor with antifouling characteristics. In recent studies, PEG and its hybrids have been employed for the fabrication of electrochemical [82,83,84,85,86,87,88,89,90], optical [91,92,93,94,95,96,97,98], and mass based [99,100,101,102] biosensors.

### 3.3. Polyacrylate Families

Polyacrylic acid, polyhydroxyethyl methacrylate, polyacrylamide, and poly(*N*-isopropylacrylamide) are some examples of stimulus-responsive hydrogels used mainly for pH and temperature sensing within a biosystem. As their hydrophilicity hinges on charged group density, ionic hydrogels can reversibly swell and de-swell relative to changes in surrounding conditions. Recently, fabrication patterning techniques have been noted as an essential development phase for greater utilization of the sensing properties of ionic hydrogels, especially when they are used without utilizing immobilized bioreceptors. Micromolding, microlithography (photomask, ion beam, and optical maskless) [103], wet-etching, microcontact printing, and evaporation-induced self-assembly are well-known rapid prototyping methods reported in some studies. Development sensing systems for taste [104], ammonia [105,106], glucose [107,108,109], pH [103,110,111,112,113,114,115,116,117,118,119,120,121,122,123,124,125,126,127,128,129], humidity [130,131,132], chemical and biochemical molecules [133,134,135,136,137,138,139,140,141,142,143], and temperature [144] have been reported using polyacrylate family hydrogels.

### 3.4. Hydrogels with Biological Origin

Polysaccharides and polypeptides including alginate, chitin and chitosan, agarose, cellulose, dextran, and hyaluronic acid are biomaterials with gel-forming properties. The unique properties of these biological origin hydrogels—including biocompatibility, hydrophilicity, heavy metal ion chelation, high protein affinity, ease of surface chemical modification granted by the availability of reactive functional groups, low cost of preparation, acceptable mechanical properties, and facile fabrication method even in small geometries—make them attractive for biosensor applications [145]. The numerous applications of biological origin hydrogel with immobilized bioreceptors reveal that they are widely utilized in the fabrication of biosensors [46,47,146,147,148,149,150,151,152,153,154,155,156].

### 3.5. Electroconductive Hydrogels

Electroconductive hydrogels, combinations of hydrated structures with electronic functionality, have attracted attention in the biomaterials field due to their unique properties. In such hydrogels, conductive polymers facilitate electron transport across the interface, while a large surface area with greater diffusivity is provided by porous hydrogels. The flexibility and processability of these hydrogels when conducting electrons, as well as functionalities conferred by chemical modifications, make them prominent in biosensors’ technology. Polypyrrole, polyaniline, and poly(ethylenedioxy thiophene) are the most common conducting polymers used for conductive hydrogel fabrication. Electrochemical enzyme-immobilized biosensors are redox active, hence their reaction with the biosystem environment results in electron transfer across the electroconductive hydrogel that produces a current or alters the potential to generate voltage. On the other hand, the doping/de-doping mechanism of conductive hydrogels leads to changes in surface resistance, current, or voltage that can be monitored to gauge response to concentrations. Also, the impedance between electrode and environment decreases due to the ionic conductivity of electroconductive hydrogels. Electroconductive hydrogels have been used for the detection of vitamins [157], glucose [158,159,160,161,162,163,164], human metabolites [165], cell viability and function [166], lactate [167,168], DNA [169,170,171], dopamine [172], peptide [173], tumors [174,175], and hydrogen peroxide [176].

## 4. Immobilization Techniques

Utilizing a reliable strategy for the immobilization of bioreceptors on a hydrogel surface is one of the most important and critical steps in biosensor design and fabrication. Selection of an appropriate immobilization strategy, monitoring of the bioreceptor’s degradation and viability, availability of reactive groups, and binding type (covalent or non-covalent) all need to be taken into account [177]. In general, bioreceptors can be immobilized on the hydrogel surface through physical adsorption and entrapment, covalent binding, crosslinking methods, or a combination of some of these techniques. Based on the technique selected, the immobilization method can be reversible or irreversible [173]. In the case of irreversible immobilization, the attached bioreceptor cannot be detached without destroying either the hydrogel microstructure or the activity of the bioreceptor.

Hydrogels that are prepared by the solution polymerization are appropriate for bioreceptor immobilization by entrapping; however, bioreceptor leakage is undesirable [178]. The entrapment method is preferable if the average intermolecular distance of the hydrogel (hydrogel porosity) is less than the size of the bioreceptor. Due to the stability of covalent bonds shaped between hydrogel and bioreceptor, covalent binding immobilization is the most widely used of the different immobilization methods [179]. However, because release of the bioreceptor upon use is minimized in this method, special attention is required to avoid blockage of the active site by the possible formation of covalent linkages. Crosslinking agents with functionalities greater than 2 are widely used in crosslinking-immobilization techniques. The method is mainly based on the ability of a crosslinker to react with a bioreceptor and the hydrogel surface.

Some costly irreversible methods are available. Affinity ligand binding and physical adsorption is a simple immobilization technique in which bioreceptors are attached to the hydrogel surface through van der Waals reactions, salt linkage, or hydrogen binding. The functionality of the bioreceptors is preserved using this technique; however, the immobilization process may be reversed by environmental conditions. The limitations of this technique are weak reproducibility, random orientation of bioreceptors attached on the hydrogel surface, and weak attachment. Chelation, which is known as metal binding, is an excellent choice for an immobilization technique when hydrogels of biological origin are employed in biosensor structures. Using a heating or a neutralization process, precipitation of a metal salt or hydroxide leads to their binding through coordination of nucleophilic groups. Depending on the mode of bond cleavage and formation and on the environment and conditions of the reaction site, unoccupied binding sites remain free for interaction with bioreceptors. Non-uniformity of the bioreceptors’ adsorption, as well as metal ion leakage, make the chelation method less reproducible; however, using chelator ligands has improved that immobilization technique. Establishing disulfide bonds between bioreceptors and hydrogels is a unique physical immobilization method. The formed stable covalent bonds tend to be broken by reacting with suitable agents or when modulation of reactivity under alteration of the solution’s ionic strength occurs.

Stabilization of bioreceptors is another important aspect of biosensor fabrication that is performed after immobilization, and it refers to shelf or operation stability. The capability of a bioreceptor to retain its activity or conformation is known as stabilization. The best-known process to provide stability to bioreceptors is the use of polyelectrolytes to increase the stability of certain enzymes and antibodies. The sugar (sugar-alcohol) component of a polyelectrolyte modifies the bioreceptor’s environment by replacing it with free water. The protecting hydration shell helps the protein to maintain both its structure and its activity. Some strategies that offer a combination of polymer and polyelectrolytes have synergic effects on the stabilization of bioreceptors [179]. Selected methods that have been proposed are lyophilization, vacuum drying, continuous culture, and encapsulation for microorganism stabilization in biosensors [177].

## 5. Transducing Strategies

As in other sensors, the transduction methods in biosensors can be classified into one of three main methods: optical, electrochemical, or mass based detection, as was indicated in Figure 1. The chemical reactions between immobilized bioreceptors and targeted biosystems that lead to electron (ion) consumption (production) form the fundamental principle for electrochemical biosensors. The rate and amount of electron consumption or production affects the measurable electrical properties (i.e., potential, current) of a biosystem. Optical transducers measure alterations in selected optical properties that occur as a consequence of changes in the biosystem’s environment. The mass measurement of minor changes is another form of transduction that has been used with biosensors. Mass change due to the binding of chemicals can be measured electrically using a mass based transducer.

**Figure 4:**
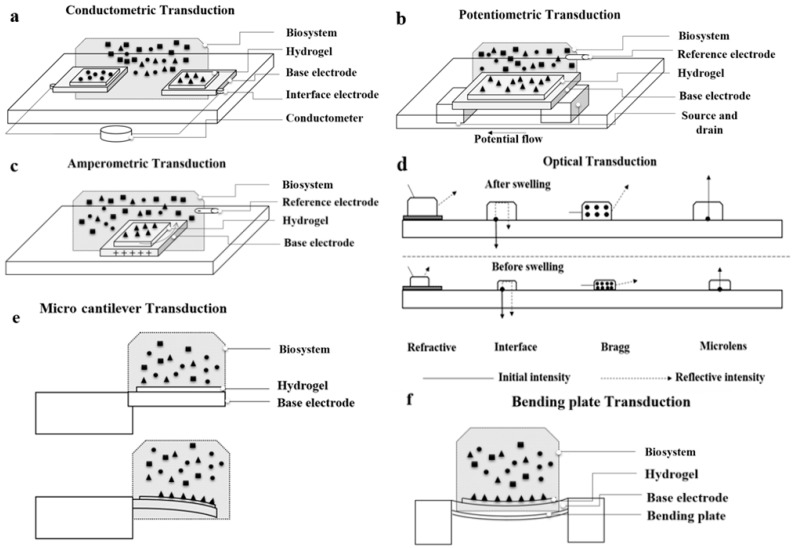
Conventional transduction methods in biosensors, including (**a**–**c**) electrochemical; (**d**) optical; and (**e**,**f**) micromechanical methods.

Electrochemically based detection techniques—including amperometric, conductometric, potentiometric, and impedimetric—as shown in Figure 4a–d respectively, are more complementary than other techniques, such as optical and mass based methods, so that hydrogel based biosensors have restricted influence in this category. With the objective of miniaturization of biosensors, pH, or electroconductive hydrogels integrated to electrode conductimetric transducers have been used for sensing glucose, monosaccharaides, and nucleotides [178,180,181,182].

As the principle of transducing methods mentioned has been well described previously, a brief review is provided here. In optical techniques, as shown in Figure 4d, detection may be based on absorption, fluorescence, and light scattering. Recognition of a biological event using hydrogel based biosensors is mainly transduced by measuring the change of fluorescence arising from hydrogel-free swelling, with high sensitivity and selectivity to low bimolecular concentration. Three types of fluorescence biosensing are direct sensing of a molecule before and after reaction, indirect sensing by transducing a dye that shows a specific biomolecule, and generation of a fluorescence signal, which is known as fluorescence energy transfer. Detection of a refractive index is another optical transduction technique that is used in preference with thin layer hydrogels coated by a metal coating. The sensitivity of the measured refractive index at the hydrogel–metal layer interface relates to metallic coating’s surface nanostructure, periodicity, and efficiency of adhesion. Changes in the reflection of incident light at hydrogel-transducer and hydrogel-bio-system edges provide another method of optical biosensing. By the embedding of a crystalline colloidal array within the hydrogel of a biosensor and by its subsequent rearrangement due to swelling/ de-swelling processes, shifts in the wavelength of diffracted lights can be measured. This method, known as Bragg diffraction, is widely used in biomedical applications.

In hydrogel based biosensors whose mechanical work is measured for biosensing, the wide range of mechanical transduction methods available includes pressure sensors, capacitive sensors, cantilever based sensors, bending plates, and microgravimetric sensors, as shown in Figure 4e,f. Pressure and capacitive biosensors are less common than other transducing methods because the multiple processing steps involved in fabrication make them expensive, and they suffer from insensitivity. In contrast, changes in mass, temperature, or stress can be transduced in both dynamic and static modes by using a microcantilever detection method. Changes in hydrogel properties that lead to swelling variation result in alteration of surface stress and bend microcantilever transducers.

## 6. Biomedical Applications of Biosensors

Numerous investigations, research studies, and innovations relating to the physical, chemical, mechanical, and biocompatibility properties of stimulus-sensitive hydrogels have contributed to our understanding of their novel potentials for biological signal sensing in medical and biomedical activities. Using biosensors to monitor physiochemical changes in the body provides opportunities for early diagnosis, treatment, and management of disease. Despite some limitations concerning accuracy, significant progress has been made in producing advanced biomaterials that facilitate a new generation of biosensor design and construction, minimizing imprecision and slow responses to physiological conditions, for enhanced therapeutic effect. As well, the successful integration of small bioreceptors with sensing components, a vital step towards miniaturization, has made the biosensing process more developed and attractive [183].

### 6.1. Cell Metabolite Detection

Cultivation of cells and intermittent cell or media culture collection are outdated methods for measuring cellular metabolism. Not only do they require many cell and reagents, but they also yield only slight information about the dynamics of metabolism and cell multiplexing. To overcome these inefficiencies of traditional methods, new biosensing approaches have been developed to monitor biosystem changes at the site of the cell. That possibility has been revealed by different research groups [184,185,186,187]. The detection of hydrogen peroxide secreted by stimulated macrophages [188], extracellular lactate [189], insulin [178,190], pH and calcium ion [191], and nitric oxide [192] has been reported in cell metabolite analyses. Currently, the key concepts for measuring cell metabolism are microfabrication techniques including photolithography [193,194,195], hybrid processes of thin-film and laminate technology [48,196,197], microcontact printing [48,198,199], microfluidic patterning and micro-channels [48,197,200,201,202], laminar flow and stencil patterning [202].

These methods have achieved high sensitivity and specificity; however, they have not been adopted in the mainstream for long-term monitoring of cell metabolism due to concerns about their stability and efficient operation in complex biosystem media and also their biocompatibility. However, covalent bonding of bioreceptors has been used to address their increasing stability in long-term applications, when the amount of immobilized bioreceptors at the cellular level is limited [203].

### 6.2. Tissue Engineering

As immobilizing scaffolds for bioreceptors, hydrogels have been employed to detect and measure the concentration of specific biomolecular interaction and kinetics in tissue engineering. With its extracellular signaling activity, adenosine triphosphate (ATP) is a vital multifunctional molecule with a key role in cellular metabolism. Recently, hydrogel based biosensors have been used for measuring ATP in situ and in vivo [204], as the application of previous methods based on luciferase has been limited by low sensitivity, resolution, and accuracy. Tissue engineers can identify a wide range of cellular activities [205,206] including cell proliferation, migration and differentiation, apoptosis, cytokine release, and necrosis by measuring extracellular ATP [207,208,209], adenosine diphosphate (ADP) [210,211], and uridine triphosphate (UTP) using biosensors [212].

The detection of targeted nucleic acids in tissue engineered scaffold has been reported using different biosensing strategies and biomaterials; however, their pre-preparation and fabrication were reported to be difficult [49,213,214,215]. Therefore, work on the integration of hydrogels of biological origin with electrochemical biosensors has recently been strongly focused on making biosensing easier and relatively inexpensive [49,216].

The fundamentals of cell biology for tissue engineering applications can be quantified by measuring the activity of functional protein molecules. For example, as a response to tissue remodeling processes, matrix metalloproteinase (MMP) that is released by cells can be used as a biomarker [217,218]. Detecting interactions between scaffold microenvironment, stem cells, and surrounding tissue is important for monitoring changes in the mechanical and physical properties of scaffolds that may lead to alteration of properties or scaffold disassembly. Labeling methods are not suitable for non-invasively monitoring scaffolds and their suboptimal biomolecular changes over time. Therefore, label-free methods are employed for real time screening of irregularity of extracellular proteins [219,220,221].

### 6.3. Wound Healing Biosensors

Wound healing can be better managed by using biosensors for early identification of wound infection and remote screening of wound factors. It is well known that during wound healing phases, inflammation and proliferation are responsible for producing exudate that contains electrolytes, MMPs, and proteins, whose concentration changes can be associated with the risk of development of wound problems [222]. Biosensor research in wound monitoring has primarily been performed for physiological indications, such as temperature, pH, and moisture [223,224,225], while uric acid in wound exudate is highly correlated with wound bacterial infection [226,227,228]. Complete approaches towards using biosensors for wound exudates to detect bacteria, temperature, oxygen, and enzymes, as well as to monitor pH, have been presented in review papers [229,230,231,232].

### 6.4. Cancer Monitoring

The sensing of two inflammatory signals—H_2_O_2_ and cytokines (TNF-α)—is important for early cancer detection. As a small molecule, H_2_O_2_ can usually be detected by enzyme based biosensors, whereas antibody based biosensors (immune-sensors) are used to detect TNF-α [233]. The imprinting capability of acrylamide hydrogels integrated with quartz crystal microbalance sensors for loading of proteins via crystallization has been used for cancer detection [234]. Poly(ethylene glycol) diacrylate hydrogel matrix that contains ferrocene-coupled superoxide dismutase has been introduced as a novel sensitive hydrogel based sensor for detection of superoxide anions released by cancer cells [193]. Ultrasensitive label-free lectin based biosensors [67], chitosan based biosensors [156], and PEG based biosensors [87,95,235] have been reported as applicable for early diagnosis of cancer. The application of novel nanoparticles (graphene, gold, silver, etc.) in combination with hyper-branched hydrophilic polymers in the immune-sensing of carcinoembryonic antigen has been discussed in detail [175] and the performance improvement of nanostructured metal oxide that could be coupled with hydrogel-based biosensors via engineering of morphology, size, interface with biomolecules, functionality, and adsorption ability has been reported [236]. Substitution of the electric cell-substrate impedance sensing method performed by monitoring specifically labeled cells, to overcome the limitations of conventional cancer detection techniques, has been used to monitor the viability, morphology, and change in environment of the adhered cells [237,238,239].

The use of molecules with defined properties as biosensors for cancer detection on the basis of their ability to bind to different targets, their chemical and thermal stability, synthesis, and storage has been explained [50].

### 6.5. Pathogen Detection

Significant efforts have been focused on the development of hydrogel based biosensors for detection of viruses [235,240,241,242]. Viruses that have been detected using hydrogel based sensors include the influenza virus [243,244,245], hepatitis B virus [246], different pathogens [13,45], and West Nile virus protein domain III [247].

### 6.6. Detection of Small Molecules

Monitoring small molecules such as glucose and cholesterol with high sensitivity and specificity offers important opportunities in primary diagnosis, early treatment, and better management of chronic disease. Table 2 indicates some specifications of hydrogel based biosensors for the detection of small molecules.

## 7. Future Outlook

Biomedical application of hydrogel-based biosensors was discussed in this study. Different hydrogels with biosensing abilities were identified. The most frequently used hydrogels are polyvinyl alcohol, polyethylene glycol, polyacrylate families, and electroconductive hydrogels. Hydrogels with biological sources include alginate, chitin, chitosan, agarose, cellulose, dextran, and hyaluronic acids. Bioreceptors to be immobilized on hydrogel-based biosensors, their advantages and disadvantages, and immobilization techniques were discussed here. Our review study showed that hydrogel based biosensors have been used for different biomedical purposes including cell metabolite and pathogen detection, tissue engineering, wound healing, and cancer monitoring, and detection strategies for small biomolecules such as glucose, lactate, urea and cholesterol. In fact, 3D swellability of hydrogels conducting electric signals, their permeability to different molecules and negligible interaction to different swelling media as well as their ability to optimal immobilization of different biomolecules suggests their roles in the future of biosensor fabrication. Some limitations including life time, storage, and adaptation with transducers for rapid quantitative analysis reveal that there is still a long way for hydrogel based biosensors to go before they are used in commercialized health management systems.

Meanwhile, the signal enhancement produced by biosensors that leads to improved measurement is conferred by labeling targeted cells. However, this process is a potential source of complexity, experimental error, and uncertainties that can place biomolecules at risk of alteration of properties. Associated costs are also involved in biosensor fabrication. Therefore, substitution of label-free biosensing strategies is essential to rendering this platform more precise, more cost effective, quicker, and more sensitive. Further important progress is required in multiple-target detection ability of the new generation of biosensors to provide wide-ranging sensing of targets in one measurement platform.

## Figures and Tables

**Table 1:** Advantages and disadvantages of different bioreceptors for hydrogel based biosensors.

Bioreceptor	Advantage	Disadvantage
Antibody [13,14,45]	The immunogen need not be purified prior to detection.	Expensive and time-consuming method. Miniaturized immune-PCR detection methods have not yet been commercialized.
Enzymes [17,18,46,47,48]	Variety of reaction products arising from the catalytic process.	Stability problems have been reported. The detection limits can be very low due to signal amplification.
Nucleic acids [49,50]	Target molecule can be recognized by shape and sequence. A wide range of biomolecules can be detected. High binding affinity, simple synthesis method and easy storage have been reported.	It is not easy to design donor/acceptor labeling strategies. They are sensitive to pyrimidine specific nucleases that are abundant in biofluids.
Cells or cellular structures [7,26,29,31]	Can be used over prolonged periods of time as cells are closed systems.	
Biomimetic [32,33,34,35,36,37]	Known as an effective, accessible and inexpensive strategy. Physically, very stable (solid-like).	Molecular imprint probes do not have the same flexibility and selectivity as actual bioreceptors.
	The molecular imprinted polymers can survive in destructive environments.	

**Table 2:** Strategies for small biomolecule detection using hydrogel based biosensors

Glucose			
Hydrogel	Transduction Strategy	Technical Specification	Ref.
Polyaniline	Electrochemical	Sensitivity = 96.1 μA·mM^−1^·cm^−2^	[248]
		Response time = 3 s	
		Linear range = 0.01–8 mM	
Polyaniline-PEG	Electrochemical	N/A	[249]
PEG	Optical	Linear range = 0–600 mg/dL	[250]
		Response time = 10 min	
PVA-Vinyl pyridine	Electrochemical	Sensitivity = 600 nA·mM^−1^·L^−1^	[251]
		Response time = 11 s	
Chitosan	Electrochemical	Linear range = 5 μM–2.5 mM	[146]
		Response time = 7 s	
Chitosan-graphene oxide	Electrochemical	Linear range = 0.02–6.78 mM	[252]
		Sensitivity = 10 μA·mM^−1^·cm^−2^	
Polypyrrole	Electrochemical	Linear range = up to 15 mM	[253]
PEG (injectable)	Optical	Linear range = up to 370 mg·dL^−1^	[254]
		Response time = 11 min	
Polyvinylpyrrolidone	Optical	N/A	[255]
Alginate	Optical	Sensitivity = 0.80 ± 0.11 μs·dL·mg^−1^	[256]
		Linear range = 2.6–350 mg/dL	
HEMA	Electrochemical	Linear range = 10 μM–40 mM	[257]
Lactate			
BH40 (Hyper-branched)	Electrochemical	Response time = 7 s	[167]
		Linear range = up to 580 mg/L	
Polycarbamoyl sulfonate	Electrochemical	Response time = 2 s	[258]
		Linear range = 10–400 μM	
Albumin-mucin	Electrochemical	Response time = 90 s	[259]
		Linear range = 0.7 μM–1.5 mM	
Chitosan	Electrochemical	Sensitivity = 0.32 A·M^−1^·cm^−2^	[260]
		Response time = 5 s	
Urea			
Polyaniline	Electrochemical	Sensitivity = 85 mA·M^−1^·cm^−2^	[261]
		Response time = 15 s	
Poly aniline	Electrochemical	Sensitivity = 878 μA·M^−1^·cm^−2^	[262]
Aniline-*co*-o-phenylenediamine	Electrochemical	sensitivity = 31.12 mV/log [M]	[263]
		Linear range = 3.16 × 10^−4^–3.16 × 10^−2^ M	
Cholesterol			
Poly(thionine)	Electrochemical	Linear range = 25–125 μM	[264]
		Sensitivity = 0.18 μA·mM^−1^·cm^−2^	
Polypyrrole	Electrochemical	Linear range = 5 × 10^−4^–1.5 × 10^−2^ M	[265]
		Response time = 30 s	
Agarose	Electrochemical	Sensitivity = 6.9 nA·μM^−1^	[266]
		Response time = 120 s	
Polyaniline	Electrochemical	Sensitivity = 0.042 μA·mg·dL^−1^	[267]
		Response time = 240 s	
Polymethacrylate	Optical	Response time = 120 s	[268]
